# Transposase-Assisted RNA/DNA Hybrid Co-Tagmentation for Target Meta-Virome of Foodborne Viruses

**DOI:** 10.3390/v16071068

**Published:** 2024-07-02

**Authors:** Danlei Liu, Zilei Zhang, Zhiyi Wang, Liang Xue, Fei Liu, Ye Lu, Shiwei Yu, Shumin Li, Huajun Zheng, Zilong Zhang, Zhengan Tian

**Affiliations:** 1 Shanghai-MOST Key Laboratory of Health and Disease Genomics, Shanghai Institute for Biomedical and Pharmaceutical Technologies, Fudan University, Shanghai 200023, China; liudanlei@fudan.edu.cn (D.L.); zhenghj@chgc.sh.cn (H.Z.); 2 Shanghai International Travel Healthcare Center, Shanghai Customs District, Shanghai 200335, China; 3 State Key Laboratory of Applied Microbiology Southern China, Institute of Microbiology, Guangdong Academy of Sciences, Guangzhou 510070, China; xueliang@gdim.cn; 4 Inspection and Quarantine Technology Communication Department, Shanghai Customs College, Shanghai 201204, China; zhangzilei@shcc.edu.cn; 5 Shandong Mental Health Center, Jinan 250014, China; 6 School of Veterinary Medicine, Kansas State University, Manhattan, KS 66502, USA

**Keywords:** whole-genome sequence, foodborne viruses, Tn5 transposase, poly(A)-negative viruses, high-throughput screening

## Abstract

Foodborne diseases are major public health problems globally. Metagenomics has emerged as a widely used tool for pathogen screening. In this study, we conducted an updated Tn5 transposase-assisted RNA/DNA hybrid co-tagmentation (TRACE) library construction approach. To address the detection of prevalent known foodborne viruses and the discovery of unknown pathogens, we employed both specific primers and oligo-T primers during reverse transcription. The method was validated using clinical samples confirmed by RT-qPCR and compared with standard RNA-seq library construction methods. The mapping-based approach enabled the retrieval of nearly complete genomes (>95%) for the majority of virus genome segments (86 out of 88, 97.73%), with a mean coverage depth of 21,494.53× (ranging from 77.94× to 55,688.58×). Co-infection phenomena involving prevalent genotypes of Norovirus with Astrovirus and Human betaherpesvirus 6B were observed in two samples. The updated TRACE-seq exhibited superior performance in viral reads percentages compared to standard RNA-seq library preparation methods. This updated method has expanded its target pathogens beyond solely Norovirus to include other prevalent foodborne viruses. The feasibility and potential effectiveness of this approach were then evaluated as an alternative method for surveilling foodborne viruses, thus paving the way for further exploration into whole-genome sequencing of viruses.

## 1. Introduction

Foodborne pathogens are prominent causes of foodborne illness with huge public health and economic consequences [1]. According to the World Health Organization (WHO), Estimates of the Global Burden of Foodborne Diseases, approximately 600 million cases of illness and 420,000 deaths in 2010 were caused by 31 foodborne hazards, including bacteria, viruses, parasites, toxins, and chemicals [2]. From 2005 to 2019, 13,374,496 cases including 534 deaths of infectious diarrhea were reported through the National Notifiable Disease Report System of China. The proportion of confirmed cases with a pathogenic diagnosis result available has increased from 1.85% to 14.29% [3]. With the development of precise detection techniques, more typical and rare pathogens have been identified and analyzed. Thus, higher requirements for the comprehensive and unbiased identification and characterization of pathogens have also been put forward.

Foodborne viruses’ outbreaks have increased in recent years [4]. The major group of foodborne viruses comprises Rotavirus, Norovirus, Astrovirus, Adenovirus, Enterovirus, Hepatitis A virus (HAV), and Hepatitis E virus (HEV) [5,6]. Beyond these, some uncommon foodborne viruses, including Salivirus and Kobuvirus, were detected during our previous surveillance on inbound population with diarrhea symptoms [7]. Meanwhile, whole-genome sequencing and phylogenetic analyses of the viral genomes are crucial to understanding the virus evolution and outbreak dynamics, with the ultimate aim of reducing the spread of the disease. A comprehensive assessment of genome-intact foodborne viruses would be highly informative for understanding mechanisms of virus transmission and evolution. However, complex molecular analysis techniques ideally based on near whole-genome sequencing of individual foodborne viruses are highly demanding. 

Next-generation sequencing (NGS) workflows utilize simultaneous and parallel techniques to amplify and sequence large amounts of genetic material [8]. The utilization of metagenomics offers the advantage of detecting unforeseen pathogens by sequencing all genetic material present in a sample, whereas targeted transcriptome sequencing excels in enriching genetic targets for specific pathogens, potentially enhancing analytical sensitivity. However, it is important to note that targeted transcriptome sequencing may miss untargeted sequences and cannot provide whole-genome sequences for pathogen epidemiological investigation. Meta-transcriptomics has a limitation that arises from substantial competition posed by the larger human genome [9]. To address this issue, the removal of ribosomal RNA (rRNA) is frequently executed prior to meta-transcriptomics, which is accomplished through poly(A)-mRNA selection or rRNA removal. Most known foodborne viruses possess a single-stranded positive-sense genomic RNA with a poly(A) tail at the 3′-terminus (e.g., Norovirus and Sapovirus in the family Calicivirus). However, some viruses initiate translation in the absence of a poly(A) tail by using functional analogues (e.g., Hepatitis C virus, Rotavirus) and will be missed when using the poly(A)-mRNA selection method [10]. Especially for Rotavirus, which possesses a segmented genome consisting of 11 gene segments and different genotypes were defined for each genome segment based on Rotavirus whole-genome sequences, requires a higher level of specificity and a broader spectrum of meta-transcriptomic library preparation.

Recommendations for metagenomes in clinical virology from the European Society for Clinical Virology (ESCV) Network mentioned that enrichment for specific viral targets using spiked primers during reverse transcription has been suggested to increase sensitivity [10]. Tn5 transposition has been widely applied to construct shotgun fragment libraries for metagenomes and altered to be capable of direct tagmentation of RNA/DNA hybrids in vitro [11,12]. Meanwhile, Tn5-mediated transposition has been confirmed to have low requirements for sample input concentration (5 ng), which is suitable for foodborne pathogen detection in laboratory and clinical samples. Previously, our team established a Tn5 transposase-assisted RNA/DNA hybrid co-tagmentation (TRACE) library construction strategy for specific viruses and evaluated it by using Norovirus clinical samples [13]. By achieving specific recognition of the Norovirus viral genome, the percentage of effective reads could be increased with the difficulty of reverse transcription caused by high GC regions can be overcome. The method developed offers a promising avenue for acquiring viral whole genomes, particularly for viruses that are challenging to culture in vitro due to low titer and complex host backgrounds. 

In this study, an updated TRACE-seq method was designed for the screening and whole-genome sequencing of foodborne viruses. We aimed to broaden target pathogens from simply Norovirus to the epidemic foodborne viruses. The viability and potential efficacy of this approach were subsequently evaluated to be an alternative method for foodborne viruses’ surveillance, offering new avenues for further exploration in this field.

## 2. Materials and Methods

### 2.1. Sample Collection

Anal swab samples were collected from the Biological Sample Bank of Shanghai International Travel Healthcare Center (Sample ID: TR) and Jinan Center for Disease Control and Prevention (Sample ID: JN). The presence of foodborne pathogens including *Salmonella* spp., *Shigella* spp., *Vibrio cholerae*, *Vibrio parahaemolyticus*, *Escherichia coli* O157:H7, Rotavirus A and Norovirus GI/GII were detected using a commercial qPCR kit (Bioperfectus Technologies, Shanghai, China) [7]. Following the qPCR results, 17 samples were chosen that tested positive for viruses and negative for bacteria. These samples were selected for further testing and comparative analysis of sequencing library preparation methods.

### 2.2. Design of Reverse Transcription Primer Pool 

Viral genomes were selected and downloaded from BV-BRC databases (bv-brc.org) and aligned using MAFFT to identify conserved regions suitable for primer design. Selection criteria for reverse transcription primers included high conservation of the target fragment region and a length ranging from 15 to 25 bp. Primers were designed using the CLC Genome Workbench to cover the epidemic foodborne viruses including Astrovirus, Coxsackievirus A6, Coxsackievirus A10, Coxsackievirus A16, Coxsackievirus B2, Enterovirus, Hepatitis A virus, Hepatitis E virus, Norovirus, Poliovirus I, Poliovirus II, Poliovirus III, Rotavirus, Salivirus and Sapovirus. The primers developed in this study were listed in Table S1. Primers were synthesized by GENEWIZ Biotech Co., Ltd. (Suzhou, China) and further configured into primer sets with a final concentration of 10 μM as per the specified requirements.

### 2.3. Nucleic Acid Extraction

Nucleic acid extraction was performed using the Magnetic Bead 96 Viral Isolation Kit (Bioperfectus Technologies, Shanghai, China) following the manufacturer’s instructions. A total of 200 μL of the sample was added to the corresponding wells in columns 1 and 7 of the reagent plate, and the corresponding label was made. Another 4 columns containing wash buffer and elution buffer separately were kept along with the sample columns and allowed for extraction. Heating elution at 70 °C helps to improve the efficiency of nucleic acid extraction. The magnetic beads air-drying time was set as 3 min to eliminate inhibitors. Nucleic acid was eluted in 80 μL elution buffer. Half the extracted RNA from each sample was used for TRACE-seq library construction and the other half for standard Illumina RNA-seq library preparation. 

### 2.4. TRACE-seq Library Preparation Based on Specific Primer Pool

Libraries were prepared using the TruePrep RNA Library Prep Kit for Illumina (Vazyme Biotech Co., Ltd., Nanjing, China, https://www.vazyme.com/product/633.html (accessed on 24 June 2024)) with some modifications. RNA (8.0 μL) was heated at 65 °C for 5 min and rapidly cooled on ice for 2 min. Subsequently, it was mixed with 2.0 μL of 5× Genomic DNA Wiper Mix and incubated at 42 °C for 2 min to eliminate genomic DNA. Then 2.0 μL 10 × RT Mix, 2.0 μL HiScript III Enzyme Mix, 2.0 μL primer sets (0.5 μL oligo-T and 1.5 μL designed primer pool), and 4.0 μL nuclease-free ddH_2_O were added to each tube for reverse transcription. Reaction conditions were as follows: 25 °C for 5 min, 37 °C for 20 min and 85 °C for 5 s. Transcription products were then mixed with 10.0 μL tagment buffer and 5.0 μL Tn5 enzyme. Tagmentation was performed at 55 °C for 15 min and held at 10 °C. Upon completion of the reaction, 2.0 μL of reaction stop solution (1% Tris (hydroxymethyl) aminomethane, 0.2% sodium lauryl sulfate, 98.8% nuclease-free ddH_2_O) was immediately added to the reaction mixture. After vortexing for mixing, the solution was incubated at 37 °C for 5 min. Then, 50.0 μL VAHTS HiFi PCR Amplification Mix, N5XX (5.0 μL) and N7XX (5.0 μL) (Index for Illumina, Vazyme, Nanjing, China) were added based on the number of samples and index matching strategy, and 1.0 μL TSE (97.394% water, 1.5% Tris (hydroxymethyl) aminomethane, 0.1% magnesium chloride, 1% potassium chloride, and 0.006% polymerase) was added into solution (37.0 μL in total). The post-cycling conditions were as follows: initial denaturation at 72 °C for 3 min, 95 °C for 3 min, followed by 15 cycles of PCR with 98 °C for 20 s, 60 °C for 15 s, 72 °C for 30 s, and a final extension at 72 °C for 5 min. The library was purified using 80.0 μL (0.8×) VAHTS DNA Clean Beads (Vazyme, Nanjing, China). Following library construction, the quantification of the library was performed using a Qubit fluorometer with the Qubit dsDNA HS Assay Kit (Invitrogen, Carlsbad, CA, USA). Library quality was confirmed by the Agilent 2100 Bioanalyzer (Agilent 2100 Bioanalyzer, Agilent, Santa Clara, CA, USA; Agilent High Sensitivity DNA Kit, Agilent, USA). Sequencing was performed using PE150 (platform: Illumina NovaSeq 6000).

### 2.5. Standard Illumina RNA-seq Library Preparation

Standard Illumina RNA-seq sequencing libraries were constructed from the extracted RNA using standard Illumina library preparation protocols. Paired-end libraries were synthesized using the TruSeq RNA Sample Preparation Kit (Illumina, San Diego, CA, USA) following the guidelines. Ribosomal RNA removal was performed with the Ribo-Zero rRNA removal kit (Illumina, San Diego, CA, USA). cDNA synthesis, end repair, A-base addition and ligation of the NGS-indexed adaptors were performed according to the protocol. Libraries were then size-selected for cDNA target fragments of 200–300 bp on 2% Low Range Ultra Agarose followed by PCR amplification using Phusion DNA polymerase (NEB, Ipswich, MA, USA) for 15 PCR cycles. All samples were sequenced in the Illumina NovaSeq 6000 platform with pair-end 150 bp (PE150) mode. 

### 2.6. Bioinformatics Analysis

Bioinformatics analysis of the NGS data was conducted using our lab bioinformatics analysis pipeline [7]. Briefly, sequence read quality was assessed using Fastp and Multiqc, followed by trimming of raw data to remove sequences with low quality using Fastp [14]. Species identification was performed using Kraken2 with the NCBI nt database (version 29 November 2023) [15], and the Kraken2 results were further analyzed using Pavian (https://fbreitwieser.shinyapps.io/pavian/ (accessed on 24 June 2024)). Metagenomic contig de novo assembly was conducted using MEGAHIT [16]. In cases where the de novo assembly results were suboptimal, Bowtie2 was employed to extract the viral sequence before assembly [17]. Taxonomic annotations were assigned to representative sequences of nonredundant gene catalogs by aligning them to the NCBI NR database using Diamond with an e-value cutoff of 1 × 10^−5^ [18]. Depth and coverage along the genome were calculated using SAMtools, and the RPKMF metric was computed as (reads per kilobase of reference genome)/(million reads passing filtering) [19]. Norovirus and Rotavirus genotyping were performed using the web-based genotyping tools https://calicivirustypingtool.cdc.gov/bctyping.cgi (accessed on 24 June 2024) and https://www.bv-brc.org/app/SubspeciesClassification (accessed on 24 June 2024), respectively [20]. 

Statistical analysis was performed using R (version 4.2). The correlation *p*-values were estimated using generalized linear models (glm). The relationships of the glm *p*-values and the correlation between the ratio of reads aligned percent, the mean coverage of the genome, the RPKMF, and the Ct value of each sample were illustrated using line plots. Differences between TRACE-seq and RNA-seq were performed as paired-sample boxplots and tested using a paired *t*-test.

## 3. Results

### 3.1. Updated TRACE-seq Experimental Methodology

**Figure 1:**
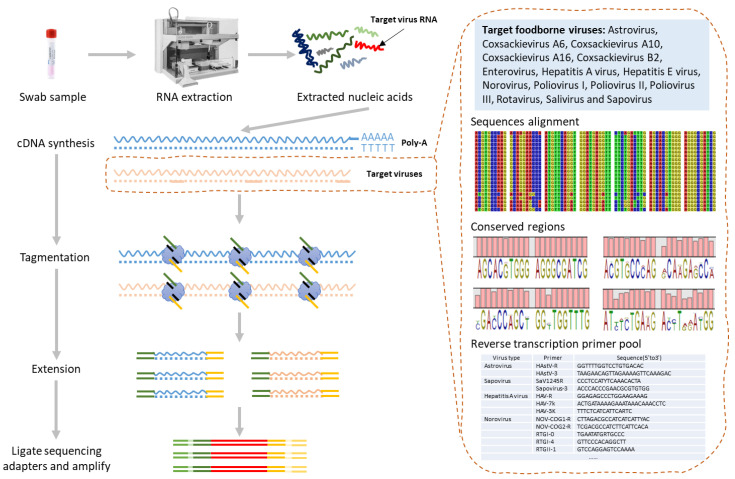
Workflow of TRACE-seq enables meta transcriptomic sequencing for foodborne virus diagnosis.

To expand the sequencing targets from Norovirus to common foodborne viruses, we updated TRACE-seq by using both specific primers and oligo-d(T) primers for reverse transcription (Figure 1). When designing primers, common foodborne viruses were taken into consideration. After aligning the sequences, conserved regions were identified, and specific primers were designed upon these regions. In addition to considering viruses with oligo-A tails, such as SARS-CoV-2, and unknown viruses, we also employed oligo-dT primers for reverse transcription simultaneously. The whole procedure, from nucleic acid extraction to a metagenomic sequencing-ready library, takes 6 h for 16 samples including waiting time. After sequencing the libraries, the initial quality control (QC) analysis was conducted, and the results indicated a successful library preparation and optimal sequencing. Statistics and quality control of the tested sequence that directly reflect the quality of library construction and sequencing were assessed with statistical methods (Table S2). 

### 3.2. Sample Information

The validation of the uploaded library preparation method primarily relies on 17 anal swab samples confirmed via RT-qPCR to be positive for nucleic acids of either Rotavirus or Norovirus (Table 1). Among these samples, nine with sample IDs starting with “TR” were collected from incoming travelers exhibiting symptoms of gastroenteritis at the Shanghai Customs port. This subset comprises six samples testing positive for Norovirus nucleic acids with cycle threshold (Ct) values ranging from 19.00 to 26.93 and three samples testing positive for Rotavirus nucleic acids, with one sample having a Ct value of 22.51, while the other two were not recorded. Additionally, eight samples with sample IDs starting with “JN” were collected from gastroenteritis patients at the Jinan Center for Disease Control and Prevention. This group includes four samples testing positive for Norovirus nucleic acids with Ct values ranging from 16.50 to 18.10 and four samples testing positive for Rotavirus nucleic acids with Ct values ranging from 15.85 to 18.53. All sample collection procedures were conducted with the patients’ consent, and informed consent forms were signed accordingly.

### 3.3. Whole-Genome Sequences Results for Updated TRACE-seq

**Figure 2:**
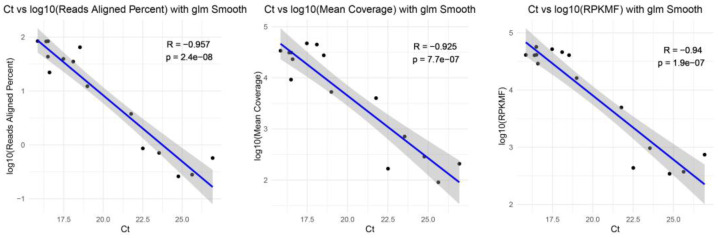
Correlation between sequencing data and Ct values. From left to right: the correlation between the ratio of reads aligned percent, the mean coverage of the genome, the RPKMF, and the Ct value of each sample. Linear regression indicates the relationship between the sequencing data and the Ct value of samples.

Given that the anal swab samples were initially confirmed to be positive for Norovirus or Rotavirus through RT-qPCR, we aimed to investigate the capability of targeted meta-transcriptomic sequencing to generate whole-genome sequences of foodborne viruses (Table 1). Even though for some samples that tested positive by RT-qPCR, TR307130012 for Rotavirus and TR306110255, TR308030002, TR310210044, TR310260051, TR311130018 for Norovirus, the relative percentage abundances of respective foodborne viruses were relatively low (0.68% to 6.62%), the mapping-based approach allowed the acquisition of nearly whole-genome sequence segments (>95%) for most virus genomes (97.73%, 86/88), with a mean coverage depth of 21,494.53× (ranging from 77.94× to 55,688.58×) and RPKMF of 28,396.67 (ranging from 209.45 to 68,862.9) (Table S3). For Norovirus-positive samples, various genotypes were identified, including epidemic subtypes GII.4 Sydney and GII.17, as well as less prevalent GII.20, GII.13, and GI.3 genotypes. The whole-genome sequences were successfully obtained, with lengths ranging from 7278 bp to 7732 bp, and a genome-wide average sequencing depth ranging from 90.40× to 47,187.66×. In the case of Rotavirus-positive samples, all samples obtained complete information for all 11 segments. The predominant genotype focused on the G3P[8] gene subtype. The mapping-based approach allowed the acquisition of nearly whole-genome sequence segments (>95%) for most viral genomes (75 out of 77), with a mean coverage depth of 22,674.22× (ranging from 77.94× to 55,688.58×) and RPKMF of 29,335.88 (ranging from 209.45 to 68,862.9). All test samples achieved reliable coverage and sequencing depth, demonstrating the robustness of this method. Subsequent correlation analysis revealed that the proportion of obtained reads of both viruses, the mean coverage sequencing depth, and the RPKMF all showed a significant negative correlation with the Ct value in all samples (Pearson test, *p* < 0.01) (Figure 2).

### 3.4. Coinfection Screening of Various Foodborne Viruses

Meanwhile, coinfection with several pathogens happens frequently in foodborne outbreaks [21]. Upon surveying the metagenomic landscape of these samples, instances of additional clinical viruses with exceptionally high abundance were observed (Table 1). For sample TR311040037, which was considered to be infected by Norovirus GII.4 Sydney[P16], Astrovirus was also confirmed. Sequencing covered the Astrovirus reference genome FJ222451.1 for 6171 bp (100%), with a mean coverage depth of 10,681.10× and RPKMF of 32,775. In the case of TR308030002, partial nucleic acid segments of human betaherpesvirus 6B were detected in addition to Norovirus GII.17[P17]. Thus, this method could not only enable the target capture of qPCR-confirmed foodborne viruses’ whole genomes but also be readily utilized to identify emerging pathogens in patients with unknown etiology of infection.

### 3.5. Comparison of Updated TRACE-seq with Standard RNA-seq Method

**Figure 3:**
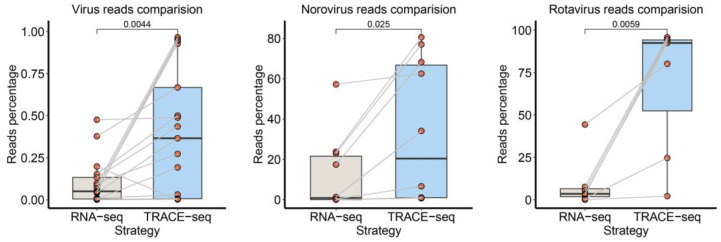
Paired boxplot of viral reads percentage comparison between TRACE-seq and RNA-seq. From left to right: the comparison in the viral reads percentage, the Norovirus reads percentage, and the Rotavirus reads percentage of each sample. Significance was compared by paired-sample *t*-tests.

To ensure the accuracy of our metagenomic computational pipeline, we employed standard RNA-seq library preparation to profile foodborne pathogens of the same samples (Table S4). The sensitivity of updated TRACE-seq was benchmarked against a previously established standard RNA-seq metric method by calculating the reads percentage of Virus, Norovirus, Rotavirus (Figure 3). Significance was assessed by conducting paired-sample *t*-tests to compare these two library preparation methods. For the viral reads percentage, updated TRACE-seq was significantly higher than the standard RNA-seq. And for targeting pathogens, updated TRACE-seq yielded a higher reads percentage and good genome-wide coverage than standard RNA-seq. Thus, as a rapid and convenient one-tube RNA-seq library construction method, updated TRACE-seq showed better performance in terms of target pathogen whole genome sequence profiling than standard RNA library preparation methods.

## 4. Discussion

Foodborne diseases resulting from the contamination of food by pathogenic microorganisms represent a significant global health concern with high morbidity and mortality rates [22]. In virology, NGS sequencing technologies have rapidly become a wonderful solution for diverse applications, including the identification of novel viruses from metagenomic samples, reconstruction of whole or nearly whole viral genome sequences, and analysis of viral evolution and species identification [23]. However, the laborious and time-consuming steps in standard RNA-seq library construction procedures hinder their clinical application. In this study, we demonstrated that targeted meta-virome TRACE-seq from anal swab samples can generate whole foodborne virus genomes with reliable depth. Due to the characteristics of Tn5 transposase, it achieves simultaneous RNA/DNA fragmentation during reverse transcription. Thus the updated TRACE-seq method is not only convenient to use and highly specific but also incorporates oligo-T primers, allowing for the sequencing of other unknown viruses. Therefore, we assert that the updated TRACE method, based on Tn5’s functionalities and characteristics, presents certain advantages over standard RNA-seq library preparation methods.

Adequate sequencing depth is crucial to ensure the reliable detection of low-abundance pathogens and low-frequency variants. When detecting viruses through metagenomic sequencing, a low horizontal coverage of the genome, with reads distributed across the genome, indicates high confidence in identifying positive virus findings. On the other hand, reads (high coverage depth) aligned at a specific part of the genome may be caused by a false positive result or the presence of a novel, distantly related virus [10]. The global multiplex PCR method has matured in its application to SARS-CoV-2 virus sequencing, significantly reducing sequencing costs. However, the high library concentration resulting from the specific amplification of two rounds of PCR in tile amplification makes it highly susceptible to laboratory aerosol contamination. Furthermore, designing primers with both sensitivity and efficiency for a broad range of strains is challenging due to sequence heterogeneity among foodborne viruses [24]. Metagenomic libraries can also be enriched for viral sequences after extraction and reverse transcription steps with capture probe enrichment methods, which are based on hybridization to a wide set of sequences specific for one or all known viruses. Although this approach mitigates the issue of excessively high library concentrations caused by targeted amplification to some extent, it significantly increases the complexity and library construction time of the experimental workflow.

In this study, an updated TRACE-seq was set up by using both specific primers and oligo-d(T) primers for reverse transcription based on the two following considerations: First, despite designing a primer pool that covers common foodborne viruses, there is a desire to employ a non-specific poly-A capture method to broadly screen and identify unknown or newly emerging pathogens. Several uncommon pathogens were identified in PCR-confirmed Norovirus or Rotavirus A positive clinical samples. Secondly, according to our preliminary experiments, low nucleic acid concentration is a recurring issue that posed challenges in the whole sequencing workflow. For clinical samples especially anal swab samples, the inherent variations in viral load between patients and different sampling operations lead to great differences in nucleic acid concentration. Additionally, due to the high specificity of primer design, the absence of oligo-T primers in some negative samples or samples with low viral content resulted in excessively low library concentrations. When the concentration levels of the library were found to be undetectable, the process of library normalization was infeasible. Thus, challenges were posed in subsequent purification and sequencing steps, rendering the assessment of on-machine data ineffective, preventing the generation of sufficient sequencing data, and ultimately hindering the success in pathogen sequencing.

Based on the testing of clinical samples, we observed that the updated TRACE-seq method, shows excellent performance in sequencing prevalent foodborne viruses, especially those without poly-A tails, such as Rotavirus. Standard RNA-seq library preparation methods are hindered by the absence of a poly-A tail, which makes it impossible to use oligo-T for capture. This limitation is particularly challenging for Rotavirus, which has a complex genome comprising 11 segments. In this study, the specific primers facilitated the complete whole-genome sequencing of Rotavirus, thereby simplifying the process. We utilized seven Rotavirus-positive clinical samples for testing and found that all 11 segments were completely obtained and successfully genotyped in each sample, demonstrating the superiority of this method. Although we included oligo-T primers alongside specific primers to account for the presence of unknown viruses, this approach means that viruses lacking poly-A tails and not recognized by our specific primers may be missed. However, our current design comprehensively covers epidemic foodborne viruses. The method of using Ribosomal RNA removal proved to be time-consuming and inefficient in practical applications, making it unsuitable for viral sequencing. Additionally, as far as we know, the primary issue with using random primers for reverse transcription is the inability to reduce background noise. However, there are teams developing specific random primers designed for eukaryotes, which is a promising development worth monitoring for future advancements.

While due to the current widespread focus on Norovirus and Rotavirus as the primary foodborne viruses, our study primarily selected clinical samples positive for these two viruses for validation. During this process, we identified two cases of co-infection: Norovirus with Human Betaherpesvirus 6B, and Norovirus with Astrovirus. Notably, Astrovirus was one of the viruses considered in our initial primer design, and the sequencing results demonstrated good performance. Our primer design included specific primers for various epidemic viruses, but we have not yet been able to validate these due to the lack of positive samples. Based on the existing results, we are optimistic about the method’s potential performance with other viruses.

Certainly, while TRACE-seq for foodborne viruses presents notable advantages, there are still some inherent limitations to consider. It’s worth mentioning that a non-random distribution of Tn5 insertion sites was evident, with certain regions exhibiting a preference for insertion [25]. However, it’s essential to emphasize that this bias did not significantly impact the results obtained from our experimental data. Moreover, the heightened enzymatic efficiency of Tn5 underscores the importance of accurately measuring nucleic acid concentration during library preparation. This may necessitate adjustments to the quantity of Tn5 input and reaction duration to ensure optimal results. Additionally, the broad range of fragment lengths resulting from enzymatic cleavage poses a challenge in achieving complete pair-end sequencing due to read length limitations. To address these challenges, we employed consensus sequences derived from comparison with a reference sequence, effectively mitigating potential sequencing issues. Furthermore, the focus of this article lies in the establishment of the updated TRACE-seq and its comparison with standard RNA-seq techniques. To achieve this objective, we deliberately selected practical samples from port monitoring and hospital testing for evaluation, based on their nucleic acid RT-qPCR detection. Consequently, there may be an issue of selecting samples with a high viral titer. In subsequent experiments, we will address this concern by testing low-copy samples. Moreover, given the proven suitability of transposase for library construction in low-concentration samples, as demonstrated in our previous studies, we have ample reason to believe in its good performance with low-copy samples. Moving forward, our future endeavors will focus on designing reverse transcription primers tailored to specific segments of foodborne bacterial genomes. This strategic approach aims to realize the goal of simultaneous detection of foodborne viruses and bacteria in a single tube. This method has the potential to significantly improve and modernize the detection process for foodborne pathogens, offering a valuable tool for enhancing diagnostic capabilities in public health and clinical settings.

## Figures and Tables

**Table 1:** Updated TRACE-seq sequencing to generate whole-genome sequences of foodborne viruses.

Sample ID	RT-qPCR	Ct	Total Filtered Reads	Reads Aligned	Reads Aligned%	Covered Bases	Mean Coverage	RPKMF *	Virus Species	Virus Genotype
TR306110255	Norovirus positive	25.63	2,270,814	6416	0.28%	7558	90.40	373.39	Norovirus	GII.4 Sydney[P16]
TR308030002	Norovirus positive	24.76	7,029,256	18,305	0.26%	7278	287.17	344.64	Norovirus	GII.17[P17]
			7,029,256	8157	0.12%	1233	3.37	7.16	Human betaherpesvirus 6B	
TR310260051	Norovirus positive	23.54	7,352,203	52,078	0.71%	7452	711.93	964.05	Norovirus	GII.20[P7]
TR311040037	Norovirus positive	19.00	2,986,176	365,863	12.25%	7567	5295.76	16,191.20	Norovirus	GII.4 Sydney[P16]
			29,86,176	603,968	20.23%	6171	10,681.10	32,775.00	Astrovirus MLB1	
TR310210044	Norovirus positive	21.77	7,978,050	299,652	3.76%	7577	4014.02	4957.05	Norovirus	GII.13[P21]
TR311130018	Norovirus positive	26.93	2,749,256	15,786	0.57%	7732	208.54	742.60	Norovirus	GI.3[P10]
JN230418N1	Norovirus positive	16.50	1,641,314	710,458	43.29%	7605	9226.61	56,917.70	Norovirus	GII.4 Sydney[P31]
JN230418N2	Norovirus positive	18.10	7,795,456	2,731,336	35.04%	7605	44,760.58	46,071.70	Norovirus	GII.4 Sydney[P31]
JN230418N3	Norovirus positive	16.60	7,488,859	1,654,221	22.09%	7605	23,139.51	29,045.50	Norovirus	GII.4 Sydney[P31]
JN230418N4	Norovirus positive	17.48	7,258,419	2,861,419	39.42%	7605	47,187.67	51,837.00	Norovirus	GII.4 Sydney[P31]
TR307130012	Rotavirus positive	22.51	2,917,878	25,102	0.86%	18,214	167.21	435.94	Rotavirus	R1-C1-M1-P[8]-A1-I1-T1-N1-G3-E1-H1
TR309140031	Rotavirus positive	-	9,764,213	6,389,046	65.43%	19,016	34,646.50	30,022.98	Rotavirus	R1-C1-M1-P[8]-A1-I1-T1-N1-G3-E1-H1
TR310080053	Rotavirus positive	-	110,570	24,478	22.14%	18,283	161.44	11,101.98	Rotavirus	R2-C2-M2-P[8]-A2-I2-T2-N2-G3-E2-H2
JN230418L1	Rotavirus positive	16.39	6,125,493	5,088,710	83.07%	18,996	31,128.48	40,607.76	Rotavirus	R1-C1-M1-P[8]-A1-I1-T1-N1-G3-E1-H1
JN230418L2	Rotavirus positive	16.51	5,735,717	4,811,697	83.89%	19,013	31,172.48	41,334.89	Rotavirus	R1-C1-M1-P[8]-A1-I1-T1-N1-G3-E1-H1
JN230418L3	Rotavirus positive	15.85	6,318,787	5,324,226	84.26%	19,043	33,894.67	41,087.07	Rotavirus	R1-C1-M1-P[8]-A1-I1-T1-N1-G3-E1-H1
JN230418L4	Rotavirus positive	18.53	5,021,832	3,254,866	64.81%	19,015	27,548.76	40,760.53	Rotavirus	R1-C1-M1-P[8]-A1-I1-T1-N1-G3-E1-H1

* The RPKMF metric was calculated as (reads per kilobase of reference genome)/(million reads passing filtering).

## Data Availability

The data reported in this study have been deposited in the Genome Sequence Archive in the National Genomics Data Center, Beijing Institute of Genomics (China National Center for Bioinformation), Chinese Academy of Sciences, under accession number CRA014353 and are publicly accessible at https://bigd.big.ac.cn/gsa (accessed on 24 June 2024).

## Supplementary Materials

The following supporting information can be downloaded at: https://www.mdpi.com/article/10.3390/v16071068/s1, Table S1: Primers list designed in this study; Table S2: Statistics and quality control of the tested sample; Table S3: Detailed information of the updated TRACE-seq method; Table S4: Detailed information of the standard RNA-seq method.
